# Electrical impedance tomography captures heterogeneous lung ventilation that may be associated with ineffective inspiratory efforts

**DOI:** 10.1186/s13054-021-03732-0

**Published:** 2021-08-21

**Authors:** Qing Pan, Mengzhe Jia, Huiqing Ge, Zhanqi Zhao

**Affiliations:** 1 College of Information Engineering, Zhejiang University of Technology, Hangzhou, China; 2 Department of Respiratory Care, Regional Medical Center for National Institute of Respiratory Diseases, School of Medicine, Sir Run Run Shaw Hospital, Zhejiang University, Hangzhou, China; 3 Department of Biomedical Engineering, Fourth Military Medical University, 169 Changle Xi Rd, Xi’an, China; 4 Institute of Technical Medicine, Furtwangen University, Villingen-Schwenningen, Germany

**Dear editor,**

Patient-ventilator asynchrony (PVA) is common in patients receiving mechanical ventilation, which is due to mismatch between neural and mechanical inspiratory time. This occurs primarily when the triggering and cycling-off of ventilatory assistance are not synchronized with the patient's inspiratory efforts. Ineffective inspiratory effort during expiration (IEE) is one of the most frequent type of PVA [1], which is associated with worse clinical outcomes [2]. Despite the extensive investigations on its mechanisms and recognition, rare notice was put on the regional ventilation distribution during IEE or other types of PVA. Electrical impedance tomography (EIT) emerges as an effective tool to monitor the regional lung ventilation [3]. In this study, we aim to describe an EIT-based method to assess heterogeneous lung ventilation, which may be associated with IEE.

Synchronized EIT and ventilator waveform were recorded in three patients with acute exacerbation of chronic obstructive pulmonary disease using PulmoVista500 and V300 (Draeger Medical, Luebeck, Germany). Patients were intubated and ventilated under assist-control mode. The relative impedance changes in ventral and dorsal regions are denoted as *I*_V_(*t*) and *I*_D_(*t*), respectively. A so-called regional intensity fraction (RIF) curve was calculated as

1 $${\text{RIF}}\left( t \right) = \frac{{{\text{Normalized}}\,I_{{\text{V}}}^{\prime } \left( t \right)}}{{{\text{Normalized}}\,I_{{\text{D}}}^{\prime } \left( t \right)}},$$

where *I′*(*t*) denotes the 1st order derivative of *I*(*t*). The normalization converts the *I′*(*t*) into the range between 0.1 and 1 for the feasibility of fractional calculation according to the following equation

2 $${\text{Norm}} \cdot I^{\prime } \left( t \right) = \frac{{\left( { I^{\prime } \left( t \right) - {\text{Min}}} \right) \times \left( {1 - 0.1} \right)}}{{{\text{Max}} - {\text{Min}}}} + 0.1$$

where $\mathrm{Max}$ and $\mathrm{Min}$ are the maximum and minimum value of *I*′(*t*). In order to examine the correlation between RIF and IEE events, the IEE events were visually inspected by a senior respiratory therapist with clinical experience > 10 years based on the stored ventilator waveforms.

We analyzed 6022 breath cycles in total. The clinical expert annotated 2945 IEE cycles and 3077 non-IEE cycles. In the expiratory phase, non-IEE cycles do not exhibit a local maximum (Fig. 1a), whereas typical IEE cycles show a distinct one (Fig. 1b). RIF_max_ indicates the peak in the expiratory phase. RIF_min_ denotes the local minimum between 0.3 s after the start of expiration and the position of RIF_max_. Delta RIF, defined as $\Delta {\text{RIF}} = {\text{RIF}}_{\max } - {\text{RIF}}_{\min }$, was associated with IEE. By selecting a threshold of 0.25 for ΔRIF, which was defined by searching the optimal value between 0.1 and 0.4 with a step of 0.05 for highest correlation between the annotation and the ΔRIF-based identification, all the 2945 IEE cycles were correctly identified as IEE by EIT. Furthermore, 920 cycles labeled as non-IEE were recognized as IEE by the proposed EIT-based method. A typical cycle with discrepant results by the ventilator waveforms and the EIT is given in Fig. 1c. Characteristic inspiratory flow was not found in the flow-time curve. However, the RIF curve shows distinct IEE features, with a significant difference between the local maximum and minimum in the expiratory phase.

**Fig. 1:**
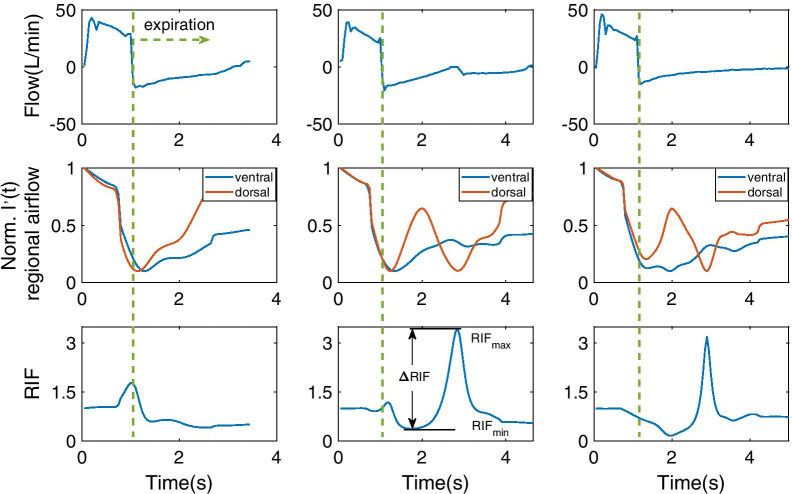
The ventilator flow-time curves (first row), the normalized 1st order derivative of regional EIT intensity (second row), and the regional intensity fraction (RIF) curves (third row) of **a** non-IEE, **b** IEE, which coincided with the characteristic inspiratory flow observed in the flow-time curve, **c** IEE, which was not able to be identified in the corresponding flow-time curve

To our best knowledge, this is the first time to uncover that a particular regional lung ventilation pattern may be associated with the occurrence of IEE. The spontaneously ineffective breath results in more imbalanced ventilation distribution, characterized by higher portion in the dorsal regions. We suspected that patient’s inspiratory effort triggered the redistribution of ventilation as proposed in previous studies [4]. However, some of these efforts were not transferred to the airway opening due to e.g. obstructive airway.

In conclusion, EIT is able to characterize the imbalanced ventilation that may be associated with IEE. It has the potential to discover IEE cycles without corresponding characteristics in the ventilator waveform. The findings require further validations with more subjects to confirm whether these cycles reflect asynchronous breath using esophageal pressure or electrical diaphragm activity, and if they will compromise the ventilatory support for the patients.

## Data Availability

The datasets used and/or analyzed during the current study are available from the corresponding authors on reasonable request.
